# RACIAL DISCRIMINATION IN HEALTHCARE SETTINGS OF OLDER ADULTS: SUBJECTIVE REASONS AND CONTRIBUTORS

**DOI:** 10.1093/geroni/igac059.1835

**Published:** 2022-12-20

**Authors:** Eun-Hye Yi, Michin Hong, Cherish Bolton

**Affiliations:** Indiana State University, Terre Haute, Indiana, United States; Indiana University, Indianapolis, Indiana, United States; Indiana State University, Terre Haute, Indiana, United States

## Abstract

Racism is prevalent in the United States; however, literature exploring racial discrimination experienced by older adults is still limited. The current study examined subjective reasons for discrimination and compared race/ethnic groups. Then, we examined the contributors to racial discrimination in healthcare settings. An older adult sample aged 55 or higher was drawn from California Health and Interview Survey 2017 for analysis (N=12,261). African Americans were the highest (13.06%) among five racial-ethnic groups who reported racial discrimination experienced in a lifetime in getting medical care, while Whites were the lowest (1.57%). Perceived reasons for discrimination were significantly different by racial/ethnic group. Only 3.5% of Whites perceived they were discriminated against due to their race, whereas racial/ethnic minorities perceived the main reason for discrimination was their race/skin color (African American: 55.43%, Others: 24.06%, Asian Americans: 20.26%, Hispanics: 18.22%). The weighted logistic regression analyses revealed that being a racial/ethnic minority, economic status, mental health status, citizenship, the length of living in the United States, and age were significantly associated with the experience of racial discrimination of older people. Analyses by race/ethnic groups found different contributors. For example, poverty was the most prominent factor in racial discrimination for Whites, while education was for African Americans. This study identified an apparent gap in lifetime discrimination toward racial/ethnic minority older people. Also, we found racial discrimination experience combined with systematic barriers. The findings of this study support the need for interventions for race/ethnicity-based trauma of older people and anti-racism framework education for healthcare professionals and researchers.

